# Morphological Changes in the Infrapatellar Fat Pad During Walking Detected by Dynamic Ultrasound in Healthy Volunteers

**DOI:** 10.7759/cureus.66738

**Published:** 2024-08-12

**Authors:** Riko Okinaka, Yosuke Ishii, Yuko Nakashima, Saeko Okamoto, Takato Hashizume, Kexin Zhu, Chen Xu, Yoshitaka Iwamoto, Nobuo Adachi, Makoto Takahashi

**Affiliations:** 1 Department of Biomechanics, Graduate School of Biomedical and Health Sciences, Hiroshima University, Hiroshima, JPN; 2 Department of Orthopaedic Surgery, Chugoku Rosai Hospital, Hiroshima, JPN; 3 Department of Clinical Practice and Support, Hiroshima University Hospital, Hiroshima, JPN; 4 Department of Orthopaedic Surgery, Graduate School of Biomedical and Health Sciences, Hiroshima University, Hiroshima, JPN

**Keywords:** knee, walking, dynamic ultrasonography, knee flexion moment, infrapatellar fat pad

## Abstract

Aim

This study aimed to verify specific morphological changes in the infrapatellar fat pad (IFP) during walking in healthy young participants.

Methods

A total of 17 healthy young participants (mean age, 22.8 ± 0.9 years) were recruited in this cross-sectional study. The IFP was evaluated using ultrasonography in three conditions: supine, standing, and walking. The IFP value was described as the thickness of the distal section of the IFP. Additionally, in the walking condition, the IFP was captured in video mode on ultrasonography, and its dynamics were recorded. The waveform of the IFP was produced using the sequence of the IFP thickness on each image. The morphological change of IFP (ΔIFP) was calculated in the IFP waveform and was shown as the difference in IFP thickness between the maximum and minimum at the beginning of the early stance phase. Moreover, kinematics and kinetic data were evaluated using a three-dimensional motion system, and the knee flexion angle (KFA) and knee flexion moment (KFM) were obtained.

Results

The thickness of the IFP during walking was significantly greater than that during the supine and standing conditions (p < 0.001 for both). The IFP waveform during walking showed a gradual increase during the stance phase and a decrease during the swing phase of the gait cycle. ΔIFP was 1.35 ± 0.42 mm and significantly correlated with the KFM (r = 0.59, p = 0.007).

Conclusions

Dynamic ultrasonography revealed a specific morphological change in the IFP during walking, which correlated with the KFM.

## Introduction

Knee osteoarthritis (OA) is a degenerative disease that causes knee pain during walking and interferes with daily activities [1,2]. Knee pain is believed to occur due to a pathological change in the knee structure that underlies mechanical stress.

The infrapatellar fat pad (IFP) within the knee joint is located between the femur, tibia, patella, and patellar tendon [3-5]. It fills the space between the patellar tendon and the tibia and moves to the posterior part of the patella with an increasing knee flexion angle (KFA). It moves anteriorly and downward with extension [3,6-8]. This flexible morphological change during joint motion plays a buffering role in the patellofemoral joint [9,10]. However, it has been shown that a decrease in the flexibility of IFP due to scarring and adhesions associated with degeneration is associated with pain [3,8,11,12]. Thus, the evaluation of the morphological change of IFP is essential for understanding knee OA pathology.

The IFP is generally evaluated using magnetic resonance imaging (MRI) [6,13]. MRI can demonstrate IFP in detail due to its ability to evaluate structures in three dimensions. However, the dynamic IFP during motion cannot be demonstrated. Ultrasonography can easily evaluate the IFP compared with MRI. The IFP is evaluated in a static state owing to the limitations of the measurement equipment, and its dynamics during walking are unknown. Currently, the internal knee structure is evaluated during walking using ultrasonography. In particular, dynamic ultrasonography can detect morphological changes in the meniscus of the knee in compartments and provide insight into knee OA [14]. It would be possible to evaluate the morphological changes in the IFP during walking using a specific transducer. It has been suggested that the morphological change in the IFP depends on a joint load [13,15,16]. The external knee flexion moment (KFM) is generated as a joint load during walking and increases the patellofemoral load [17]. Therefore, it is important to understand the morphological change of the IFP during walking that causes joint loads, such as the KFM.

It is necessary to understand the dynamics of normal IFP in healthy participants to examine the dynamic IFP in OA in the future. Therefore, this study aimed to verify whether specific morphological changes in the IFP could be evaluated during walking in healthy young participants. We hypothesized that dynamic ultrasonographic evaluation can depict the IFP during walking and that morphological changes in the IFP are different than in the static state and correlated with KFM.

## Materials and methods

Participants

A total of 20 healthy young participants were recruited (mean age, 22.8 ± 0.9 years; body mass index, 21.4 ± 2.3 kg/m^2^; female, n = 12). The exclusion criteria were a history of surgical treatment of the knee, trauma to the lower extremities, and neurological disorders. This study was approved by the Ethical Committee for Epidemiology of Hiroshima University (E2021-2498-02). All participants provided informed consent to participate in this study.

Motion analysis

**Figure 1:**
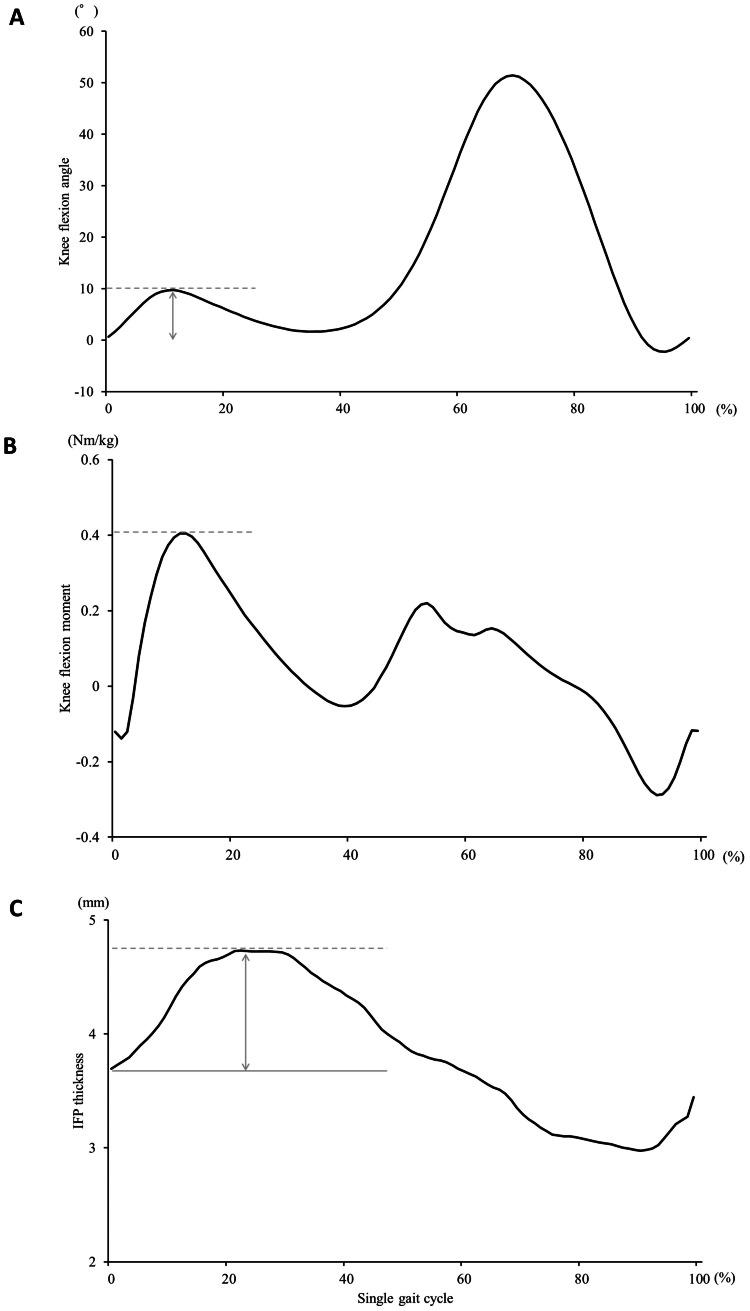
Data during one gait cycle (A) Waveform of the knee flexion angle. The waveform shows the average of all participants (dashed line, first peaks during the stance phase; arrow, the range of the knee flexion angle); (B) waveform of the knee flexion moment. The waveform shows the average of all participants (dashed line, maximum value); (C) waveform of the infrapatellar fat pad (IFP) thickness. The waveform shows the average of all participants. Line, minimum value; dashed line, maximum value; arrow, range of IFP thickness change.

Kinematics and kinetic data during gait were captured using a 16-camera motion analysis system (Vicon Motion Systems, Oxford, United Kingdom) at a frequency of 100 Hz combined with eight force plates (AMTI, Watertown, Massachusetts) at a sampling frequency of 1,000 Hz. In this study, a Vicon Plug-In-Gait lower-body model was adopted, and 16 passive reflective markers were placed on the landmarks of each participant's body. The examiner first determined the axes of each participant's joints in a static position, after which the participants were instructed to walk 5 m three times at a comfortable speed. Data analyses were performed using MATLAB R 2020a (MathWorks, Natick, Massachusetts). The analysis consisted of a single gait cycle. A single-stance phase was defined as the cycle between heel contact and toe-off, and a single-swing phase was defined as the cycle between toe-off and the next heel contact. These events were defined using a 20 N threshold on the vertical ground reaction force (GRF). A single gait cycle was normalized to 100 data points. KFA, KFM, GRF, and gait speed were calculated. The flexion angle during the gait cycle exhibited a bi-peak. The first peak was the maximum value during the stance phase, and the minimum value was set at the time of heel contact. The difference between the maximum and minimum values was defined as the range of the KFA (Figure 1A). The peak of the KFM during the stance phase was the maximum value (KFMmax), and the KFM impulse was calculated as the integral of the stance phase of the KFM (Figure 1B). KFM was normalized to body weight to account for morphological differences among participants.

Evaluation of the IFP thickness

**Figure 2:**
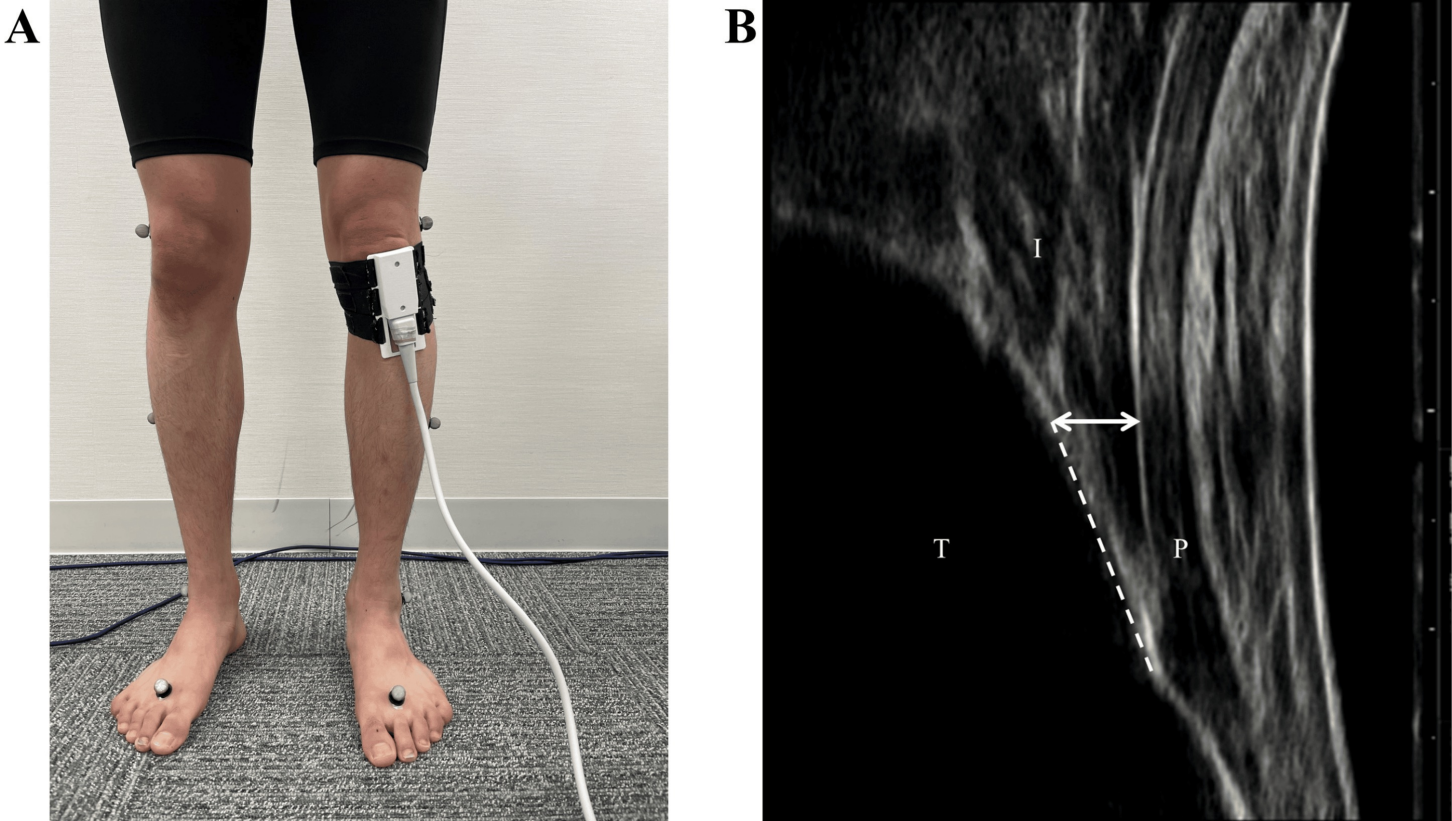
Transducer and ultrasound images (A) Snapshot of a transducer fitted to the anterior surface of the knee; (B) ultrasound image of the infrapatellar fat pad. I: infrapatellar fat pad; T: tibia; P: patellar tendon; dashed line: 10 mm line on a tibial cortex from the tendon insertion; arrow, IFP thickness.

The IFP was taken under walking and static conditions. Static condition was supine and standing with full knee extension. Ultrasound imaging of the IFP during each task was performed using an ultrasound device (SNiBLE, KONICA MINOLTA, Tokyo, Japan) with a prototype 3-11 MHz special linear-array transducer (KONICA MINOLTA, Tokyo, Japan) without special manipulation of gain operation. The focus area on the image was adjusted to be depicted clearly. A longitudinal transducer was fitted to the anterior surface of the knee along the patellar tendon (Figure 2A). The IFP thickness was measured using the Kinovea software (v0.8.15; Kinovea open-source project, https://www.kinovea.org) (Bordeaux, France). It was made with a vertical line to the undersurface of the patellar tendon, extending from the tendon to a tibial cortex point 10 mm proximal to the tendon insertion, according to a previous study (Figure 2B) [15].

In the walking condition, the participants walked with a transducer fixed to a belt. The ultrasound was performed acquired at a frequency of 30 Hz. The motion analysis system and ultrasound were temporally synchronized by an external electrical signal, and the starting time between these devices was determined. Approximately 30 ultrasound images were acquired during each gait cycle. The dates were normalized to 100 data points. The maximum value during a single gait cycle (IFPmax) was calculated, and the minimum value (IFPmin) was calculated in the range from the start of the analysis to IFPmax. The difference between the IFPmax and the IFPmin was defined as the morphological change of IFP (ΔIFP) (Figure 1C). To evaluate the reliability of IFP thickness, intra- and inter-rater reliabilities were analyzed using the intraclass correlation coefficient (ICC). Two examiners obtained additional data twice from eight participants. One examiner (R.O.) obtained ICC (1,3), and ICC (2,3) was obtained by two examiners, R.O. and Y.I., who were blinded to the participant's information.

Under static conditions, the transducer was fixed by the examiner's hands to adjust for the appropriate images. This measurement was demonstrated in supine and standing positions, with full knee extension.

Statistical analysis

For all data, the average of three trials was used for the statistical analysis, which was performed using IBM SPSS Statistics for Windows, Version 23 (Released 2015; IBM Corp., Armonk, New York). Variables were tested for normality using the Shapiro-Wilk test. The IFP thickness data were compared among the three conditions using repeated-measures analysis of variance with post-hoc Bonferroni correction. In this comparison, the IFPmax was used for the walking condition. Pearson's correlation analysis was performed to investigate the correlations between the three conditions. In addition, Pearson or Spearman correlation analysis was performed to investigate the correlation between the IFP thickness data and kinematic and kinetic data. The level of significance was set at p < 0.05.

To detect the correlation between the ΔIFP and KFM in the current sample size, the post-hoc test as power analysis was performed by G-power and showed that the statistical power was 86.2%.

## Results

Comparison of the IFP thickness among conditions

**Figure 3:**
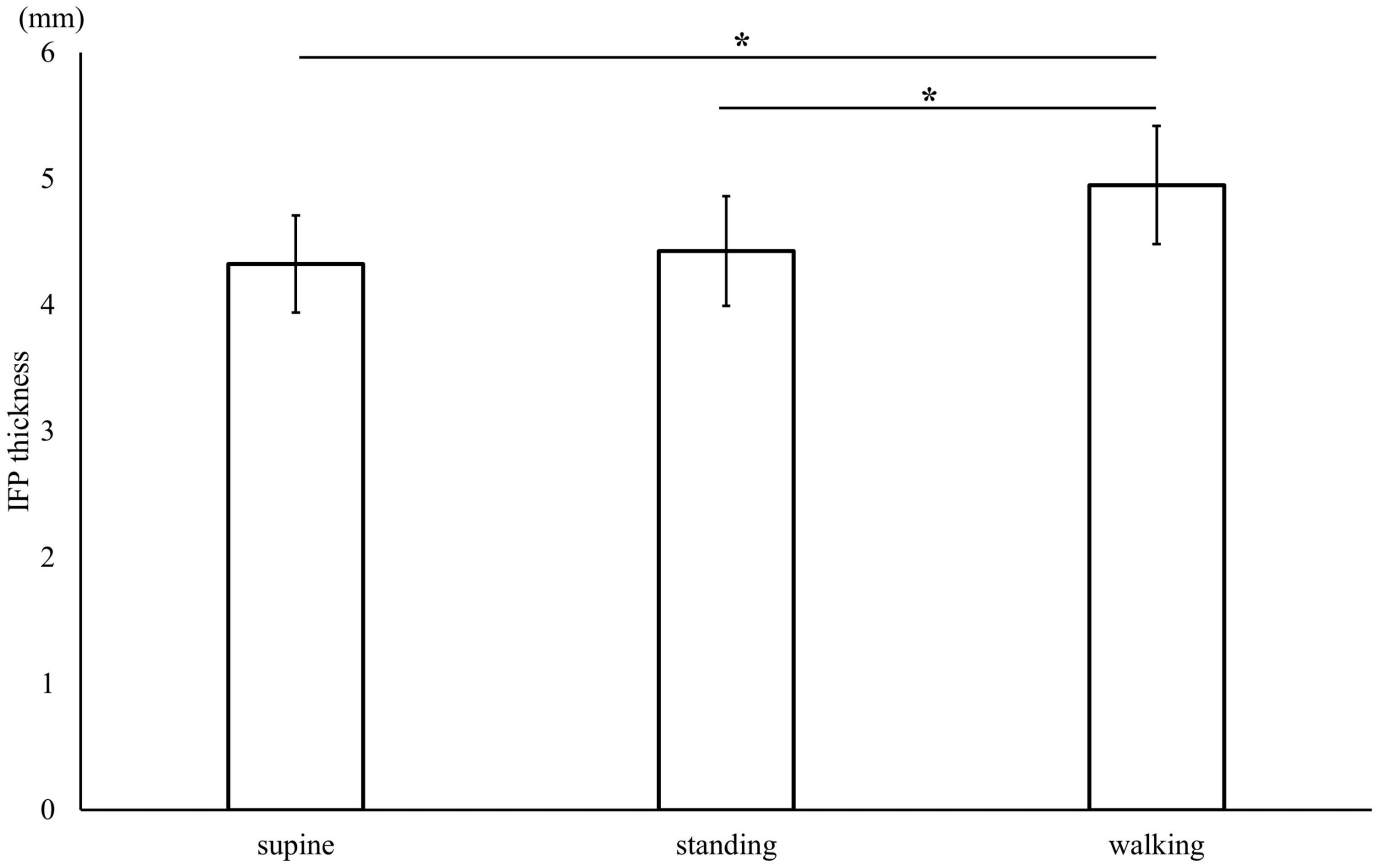
Comparison of the infrapatellar fat pad (IFP) thickness under three conditions *shows significant differences between conditions (p < 0.01). Values represent means ± standard deviations.

No significant differences were observed between the IFP thickness of supine and standing conditions. The maximum value during the dynamic condition was significantly increased compared with the IFP thickness in the supine and standing conditions (p < 0.001 for both) (Figure 3).

In addition, the IFP thickness in the supine condition was significantly correlated with that in the standing condition (r = 0.63, p = 0.003). IFPmax during walking was also significantly correlated with IFP thickness in the supine condition (p = 0.01) and the standing condition (p < 0.01). However, ΔIFP of the dynamic condition was not correlated with the IFP thickness in the supine and standing conditions (Table 1).

**Table 1:** Correlation between three conditions The r values represent the correlation coefficients of the infrapatellar fat pad (IFP) thickness during the dynamic condition with supine and standing conditions. IFPmax, the maximum value of the IFP thickness change during walking; ΔIFP, the range of the IFP thickness change during walking.

	Supine		Standing	
	r	p	r	p
IFPmax	0.55	0.013	0.81	0.01
ΔIFP	0.11	0.646	0.22	0.363

Evaluation of the IFP thickness during the walking conditions

Figure 1C shows an average of the IFP thickness during a single gait cycle. The maximum value occurred during the first half of the stance phase. The IFPmax was 4.95 ± 0.47 mm, the IFPmin was 3.60 ± 0.52 mm, and the ΔIFP was 1.35 ± 0.42 mm. The IFPmax was shown at approximately 21% of a single gait cycle. The KFM reached a maximum value of approximately 10%, corresponding to IFPmax immediately afterward (Figure 1BC). IFP thickness decreased with increasing KFA during the swing phase (Figure 1AC).

Reliability of measurement of the IFP thickness

ICC (1,3) and ICC (2,3) were 0.942 and 0.890, respectively, in terms of the maximum IFP thickness during walking. ICC (1,3) and ICC (2,3) were 0.930 and 0.737, respectively, in terms of the minimum IFP thickness during gait. ICC (1,3) and ICC (2,3) were 0.857 and 0.602, respectively, in terms of ΔIFP.

Kinetics and kinematics data and gait speed

Figure 1A shows an average of the KFA. The KFAmax was 9.94 ± 5.05°, the KFAmin was 0.28 ± 3.96°, the range of KFA was 9.66 ± 3.17°. Figure 1B shows an average of the KFM. The KFMmax and the KFM impulse were 0.44 ± 0.22 Nm/kg and 0.09 ± 0.08 Nm･s/kg. The first and second peaks of GRF were 605.28 ± 110.55 N and 638.83 ± 96.34 N. The gait speed was 1.02 ± 0.14 m/s.

Correlation between the IFP thickness and biomechanical data of the walking condition

ΔIFP was significantly correlated with the KFMmax (r = 0.59, p = 0.007) (Table 2). However, ΔIFP was not correlated with the maximum value and the range of the KFA.

**Table 2:** Correlation between the IFP thickness and biomechanical data during walking The r values represent the correlation coefficients. IFPmax, the maximum value of the infrapatellar fat pad (IFP) thickness change; ΔIFP, the morphological change of the IFP; KFMmax, the maximum value of the knee flexion moment; KFM impulse, the integral of the stance phase of the knee flexion moment; KFAmax, the first peak of the knee flexion angle during the stance phase; Range of KFA, the range of the knee flexion angle during the stance phase.

	KFMmax		KFM Impulse		KFAmax		Range of KFA	
	r	p	r	p	r	p	r	p
IFPmax	0.14	0.564	0.003	0.989	0.19	0.413	0.49	0.029
ΔIFP	0.59	0.007	0.43	0.056	0.27	0.248	0.25	0.296

## Discussion

To the best of our knowledge, this is the first study to evaluate the IFP during walking using ultrasonography, which showed a unique waveform. The IFPmax on the waveform was observed in the first half of the stance phase, and KFMmax was significantly correlated with ΔIFP, supporting our hypothesis.

The IFPmax in the walking condition was significantly greater than those in the supine and standing conditions. The IFP moves anteriorly and downward when the knee joint is extended [3,6-8]. Since the knee joint was fully extended in the supine and standing conditions but flexed during walking, the IFP thickness was expected to be thicker in both the supine and standing conditions than in the walking condition. However, the IFPmax in the walking condition was thicker than that in either of the other two conditions, suggesting that conventional evaluation methods may underestimate the dynamics of the IFP according to mechanical stress. Previous studies have compared the IFP only under static conditions [7,11,13,15], although knee pain occurs under mechanical stress during daily activities such as walking. Therefore, this study suggests the necessity to evaluate under not only static conditions but also dynamic conditions during walking. Moreover, the morphological changes in the IFP during walking did not correlate with the thickness of the IFP observed in the supine and standing conditions. Therefore, the morphological change in the IFP during walking is a unique factor that could provide insights for understanding the structural reaction under mechanical stress.

In general, the IFP thickness decreases with increased KFA [13]. However, IFP thickness increased during the early stance phase when the KFA increased. Previous studies have reported that the patellar tendon straightens and the IFP thickness increases because of quadriceps muscle contraction [15]. It is inferred that the quadriceps muscle contraction related to KFM contributes to morphological change in the IFP during the first half of the stance phase. The maximum IFP thickness during walking was observed in the first half of the stance phase. The loading response in the first half of the stance phase was one of the highest-load phases. It has been reported that shock-absorbing properties are required [17]. In this study, the KFMmax was observed during the loading response, and the IFP thickness was observed at its maximum value immediately after the KFMmax, indicating a significant correlation between the KFMmax and ΔIFP. Furthermore, the patellofemoral joint stress calculated from the joint moments was high during the loading response [18,19]. The patellofemoral joint stress is believed to increase because the KFM increases during the loading response. The dynamic IFP during the stance phase would be related to the joint load and quadriceps contraction rather than to the KFA. In healthy young participants, morphological changes in the IFP may reduce the load during walking.

Our data revealed a unique waveform of IFP thickness during walking that differed from the IFP parameters, which were evaluated only in the static state [11,13]. Moreover, dynamic evaluation demonstrated good reliability. These results indicate that dynamic ultrasound is a clinical tool for detecting morphological changes in the IFP associated with IFP dysfunction under mechanical stress. In the future, evaluation of the IFP during walking in patients with OA may provide information to determine whether the buffering effect is functioning as compared to healthy participants. The causes of OA pain differ among individuals [20]. Particularly, the poor morphological change of IFP is often observed in symptomatic knee patients under weight-bearing situations whereas the mechanism is still unknown. Evaluating the IFP during walking may be the first step to describe the symptomatic knee OA and contribute to the elucidation of the pathophysiology of clinical problems in patients with knee OA.

This study had several limitations. First, evaluating the IFP may have been influenced by muscle contraction even in the static state, Particularly, the patellar tendon tension, which depends on quadriceps muscle contraction, affects the IFP thickness in a static state [15]. Moreover, muscle contraction is easy to change under a variety of positions and joint angles. These could emphasize the evaluation of muscle activity according to the IFP morphological change and affect the directory of our findings. Second, we could not determine how muscle activity during walking affected the IFP. The change in the IFP thickness during walking is believed to be related to quadriceps muscle contraction; however, we were unable to examine this effect in this study. Therefore, it is necessary to examine the muscle activity in the future. Third, this study could not evaluate the entire IFP. Only the distal portion of IFP was evaluated in this study because of technical limitations in using the ultrasound device. However, a previous study showed that the change in the IFP is greater in the distal portion rather than in the proximal portion [21]. Therefore, the distal portion of IFP was considered to contribute to elaborating the mechanism of IFP movement. Fourth, our data did not obtain quality data on IFP such as fibrosis situations, so it remains unclear whether it affects the morphological change. However, healthy young subjects were recruited in this study, so it might have minimized the effect of IFP fibrosis on the morphological change. However, further studies are required to determine the quality of the IFP information and muscle activity.

## Conclusions

This study was demonstrated to evaluate the IFP during walking using ultrasonography. Dynamic ultrasonography revealed a specific morphological change in the IFP associated with the KFM during walking. This data could contribute to understanding the elucidation of the pathophysiology of clinical problems in patients with knee OA.
